# Impact of iterative model reconstruction combined with dose reduction on the image quality of head and neck CTA in children

**DOI:** 10.1038/s41598-018-30300-4

**Published:** 2018-08-22

**Authors:** Bochao Cheng, Haoyang Xing, Du Lei, Yingkun Guo, Gang Ning, Qiyong Gong, Wu Cai

**Affiliations:** 1 Department of Radiology, West China Second University Hospital, Sichuan University, Chengdu, China; 2 Huaxi MR Research Center, Department of Radiology, West China Hospital of Sichuan University, Chengdu, China; 3 College of Physical Science and Technology, Sichuan University, Chengdu, China; 4 Department of Psychosis Studies, Institute of Psychiatry, Psychology & Neuroscience, King’s College London, London, UK; 5 Key Laboratory of Birth Defects and Related Diseases of Women and Children (Sichuan University), Ministry of Education, Chengdu, China; 6 Department of Radiology, Second Affiliated Hospital of Soochow University, Suzhou, China

## Abstract

This study aimed to evaluate the imaging quality of head and neck computed tomographic angiography (CTA) in pediatric patients at a lowered radiation dose by combining an iterative model reconstruction (IMR) with low voltage scanning. Eighty-three pediatric patients were randomized into two groups as follows: Group A (n = 42), 100 kV/50 ml contrast media (CM), using filtered back projection (FBP); and Group B (n = 41), 80 kV/30 ml CM, using IMR. The enhanced CT value of the arteries, the image noise, the signal-to-noise ratio (SNR)/contrast-to-noise ratio (CNR), the image quality, the effective radiation dose (ED) and the iodine intake were compared between the two groups. The mean ED and iodine intake of group B were reduced by 69.8% and 40.0%, respectively, compared to those of group A. The mean CT values of the arteries in group B were higher than those in group A (*p* < 0.01), whereas the image noise of group B was lower than that of group A (*p* < 0.01). Group B exhibited a better image quality and a higher mean CNR/SNR than that of group A (*p* < 0.01). Compared to FBP, IMR in head and neck CTA enables a significant reduction in the radiation dose while preserving the diagnostic image quality. Thus, IMR, combined with low tube voltage scanning, provided an excellent solution for improving the image quality of craniocervical vessels in children.

## Introduction

With advances in multislice computed tomography (MSCT) and CT angiography (CTA) techniques, CTA of the brain-supplying arteries is frequently used in clinical practice as a noninvasive examination, especially in patients with head and neck vascular disease^1,2^. However, the radiation safety and the risk for special diseases, such as contrast agent-induced nephropathy and eye lens and thyroid gland impairment, has attracted increasing attention^3^. For children, this concern is amplified because children are more sensitive to the effects of ionizing radiation^4,5^. Therefore, under the clinical diagnostic requirements, a better strategy to reduce the radiation dose and contrast media (CM) intake in pediatric patients is the top priority in current CTA research^3,6^.

As such, a substantial emphasis is placed on strategies to reduce CT radiation doses while maintaining the image quality^7^. Some techniques, such as an automatic adjustment of the tube current^8^, a reduced tube voltage^9^, a noise reduction filter^10^ and a higher pitch^11^, have been applied in the clinical practice. However, these strategies cause increased image noise or/and decreased image quality^12,13^. In the last decade, the extensive use of the traditional filtered back projection (FBP) reconstruction technique has provided lower radiation doses than those of traditional CTA but with a limited image quality to balance the spatial resolution and image noise^14^. Lately, iterative reconstruction (IR) methods, such as adaptive statistical iterative reconstruction (ASiR)^15^ and developed model-based iterative reconstruction (MBIR)^16^, have been developed to pursue stable image quality in low-dose CT examinations.

ASiR uses complex noise statistical models, which combine filtered FBP images with iteratively reconstructed images to reduce noise and to improve image quality. Unlike ASiR, MBIR is a fully iterative method, which takes not only the data statistics into account but also the optics of the scanner, including the focal spot and detector size. In particular, to enhance the model precision of the CT scanner, MBIR utilizes complex mathematical formulations to account for physical effects, such as beam hardening, scatter and metal attenuation artifacts. Unfortunately, ASiR induces artifacts in the conjunction texture^17,18^, while MBIR induces a seriously time delay in the reconstruction process compared to FBP and ASiR, where approximately 3–4 datasets are reconstructed per hour^19^.

Recently, a newly developed knowledge-based IR, known as iterative model reconstruction (IMR), which is another fully iterative method, represents the latest advance in the field of reconstruction techniques and has the potential to reduce the image noise and improve the image quality^20^. IMR uses a knowledge-based approach to accurately determine the data, image statistics and system models of CT scanner and produces optimal images by iterative minimization of the difference between measured raw data and the estimated image via a penalty-based cost function^21^. A previous coronary CTA study utilized low tube voltage combined with IMR, which remarkably reduced the image noise and improved the image quality, indicating a potential for further dose reduction^21^. However, until recently, no study has reported the application of this method in head and neck CTA for pediatric patients. Thus, we aimed to evaluate the image quality and diagnostic performance improvement using the knowledge-based IMR method combined with a low tube voltage and a low iodine load in head and neck CTA for pediatric patients.

## Results

### Patient General Information

A total of 83 patients met our inclusion criteria and were enrolled in our study, including 42 males and 41 females. Five patients with hemorrhage/infarction following surgical procedures and three patients with serious movement during the CT scanning were excluded from our study. There were no significant differences between the two groups with regard to sex, age, body mass index (BMI), scanning length and hyoid level maximum diameter (Table 1).

**Table 1:** Patient characteristics and radiation doses among the two groups.

Characteristics	Group A (n = 42)	Group B (n = 41)
Sex (male/female)	21/21	21/20
Age (y)	14.4 ± 3.8	14.7 ± 3.5
BMI (kg/m^2^)	19.3 ± 2.8	19.2 ± 3.1
Tube voltage (kV)	100	80
Tube current (mAs)	150	150

^*^Significant differences between the group comparison; *Abbreviations:* BMI, body mass index.

### Radiation Dose

The CM administered to group B was reduced by 40% compared to that of group A. The mean CT dose index (CTDIvol, mGy) and dose length product (DLP, mGy·cm) was 19.9 ± 1.3 mGy and 897.0 ± 43.2 mGy·cm in group A, respectively, and 6.0 ± 1.1 mGy and 270.4 ± 11.7 mGy·cm in group B (Table 2).

The mean iodine load and iodine delivery rate (IDR, mGy·cm) was 19.9 mGy and 897.0 ± 43.2 mGy·cm in group A, respectively, and 6.0 mGy and 270.4 ± 11.7 mGy·cm in group B (Table 2).

**Table 2:** Radiation dose, iodine load and iodine delivery rate obtained between group A and group B.

Variable	Group A	Group B	*P*
Maximum transverse neck diameter (cm)	16.2 ± 1.3	16.3 ± 1.1	0.231
Scan length (cm)	35.0 ± 2.2	35.0 ± 2.0	0.971
DLP (mGy·cm)	897.0 ± 43.2	270.4 ± 11.7	<0.001
ED (mSv)	3.77 ± 0.13	1.14 ± 0.04	<0.001
CTDI_vol_ (mGy)	19.9	6.0	—
Iodine load (gI)	15.0	9.0	—
Iodine delivery rate (gI/s)	1.50	1.50	—

*Abbreviations:* CTDIvol, volume computed tomography dose index; DLP, dose length product; ED, effective dose.

**Figure 1:**
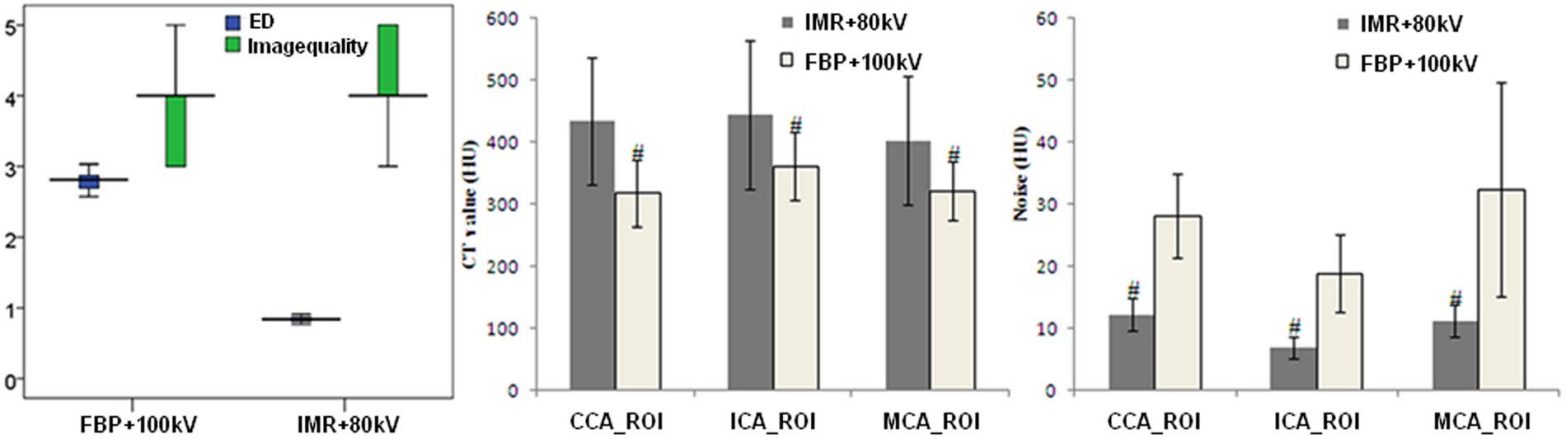
Boxplot and bar charts to visually show the improvements of the IMR algorithm versus FBP algorithm. Boxplot a show the mean ED and image quality of the IMR group versus the FBP group. Compare to FBP + 100 kV algorithm, patients received IMR + 80 kV algorithm have less radiation dose, with improvement in image quality, simultaneously. The middle chart and the right chart show the mean CT values and mean image noise in the ROIs of both groups. The CT values of the ROIs of the FBP group were significantly lower than those of the IMR group (^#^p < 0.01), whereas the image noises were significantly lower in the IMR groups than those in the group FBP (^#^p < 0.01). Error bars represent 95% confidence intervals median value and the upper and lower bars represent the first and third quartiles respectively, whiskers represent the 95% confidence interval. Abbreviations: ED, effective dose; FBP, filtered back projection; IMR, iterative model construction; ROI, region of interest; CCA, common carotid artery; ICA, internal carotid artery; MCA, middle cerebral artery.

The iodine loads in groups A and B were 15.0 gI and 9.0 gI, respectively. The IDR for groups A and B were both 1.50 gI/s. The effective dose (ED, mSv) and iodine load in group B were reduced by 69.8% and 40.0%, respectively, compared to that in group A (Table 2). The differences of the maximum transverse neck diameter and the scan length between the two groups were not significant (Table 2). There were significant differences in the DLP and ED between the 2 patient groups (*p* < 0.001, Fig. 1a).

### Image quality

#### Objective image analysis

The vascular mean CT value, image noise, signal-to-noise ratio (SNR) and contrast-to-noise ratio (CNR) of the three regions of interest (ROIs) between the two groups are listed in Table 3. The mean CT value of the common carotid artery (CCA) and the origins of the internal carotid artery (ICA) and the middle cerebral artery (MCA) were higher in group B than that in group A (pCCA < 0.01, pICA < 0.01, and pMCA < 0.01) (Table 3, Fig. 1), whereas the image noise was lower in group B than that in group A (pCCA < 0.01, pICA < 0.01, and pMCA < 0.01) (Table 3, Fig. 1).

**Table 3:** Objective image quality evaluation.

Variables	Group A	Group B	*P*
ROI1 (CCA)			
CT value of the CCA (HU)	316.9 ± 53.0	433.4 ± 101.8	<0.01
Image noise (HU)	28.1 ± 6.8	12.1 ± 2.6	<0.01
SNR	11.9 ± 3.2	37.7 ± 13.3	<0.01
CNR	10.2 ± 2.9	32.6 ± 13.3	<0.01
ROI2 (ICA)			
CT value of the ICA (HU)	359.5 ± 55.2	443.5 ± 120.1	<0.01
Image noise (HU)	18.8 ± 6.2	6.8 ± 1.7	<0.01
SNR	21.5 ± 8.7	69.7 ± 27.4	<0.01
CNR	18.0 ± 7.3	62.7 ± 26.4	<0.01
ROI3 (MCA)			
CT value of the MCA (HU)	321.1 ± 47.2	401.6 ± 104.0	<0.01
Image noise (HU)	32.3 ± 17.2	11.2 ± 2.7	<0.01
SNR	12.4 ± 5.6	39.3 ± 18.4	<0.01
CNR	10.7 ± 4.7	36.4 ± 17.7	<0.01

The data are the means ± standard deviations. *Abbreviations:* SNR, signal-to-noise ratio; CNR, contrast-to-noise ratio; CCA, common carotid artery; ICA, internal carotid artery; MCA, middle cerebral artery.

The mean CT values of the CCA, the ICA and the MCA were 316.9 ± 53.0 HU, 359.5 ± 55.2 HU and 321.1 ± 47.3 HU in group A and were 433.4 ± 101.8 HU, 443.5 ± 120.1 HU and 401.6 ± 104.0 HU in group B (Table 3). In group A, the mean CNR was 10.2 ± 2.9 for CCA, 18.0 ± 7.3 for ICA and 10.7 ± 4.7 for MCA. In group B, the mean CNR was 32.6 ± 13.3 for CCA, 62.7 ± 26.4 for ICA and 36.4 ± 17.7 for MCA (Table 3). The SNR measurements for both groups were compared as well (Table 3).

#### Subjective image analysis

**Figure 2:**
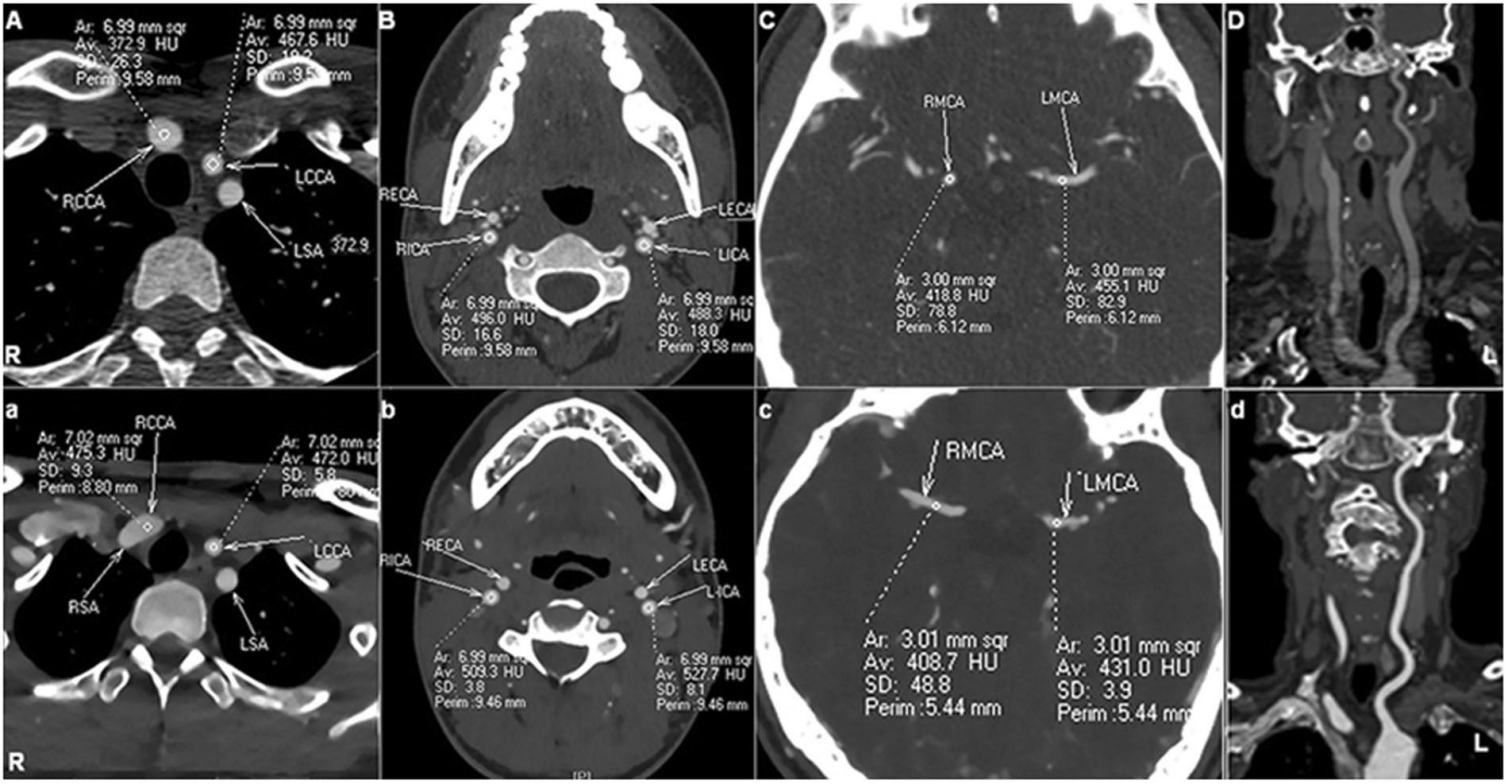
Measurements of the objective image quality in the head and neck of two 12-year-old boys reconstructed with the FBP algorithm and the IMR algorithm. The ROIs for the CT values and noises were measured for both FBP/IMR groups in the origin of the bilateral CCA (**A/a**) and ICA (**B/b**) and the bilateral M1 segments of the MCA (**C/c**). The CPR is shown in column (**D/d**). The CT values of the ROIs of the IMR group were significantly higher (566.8, 546.2 and 531.1 HU) than those of the FBP group (319.8, 356.9 and 295.1 HU) (*p* < 0.001), whereas the image noises were significantly lower in the IMR groups than those in the group FBP (*p* < 0.001). *Abbreviations:* FBP, filtered back projection; IMR, iterative model construction; ROI, region of interest; CCA, common carotid artery; ICA, internal carotid artery; MCA, middle cerebral artery; CPR, curved planar reconstruction. R, right; L, left.

**Figure 3:**
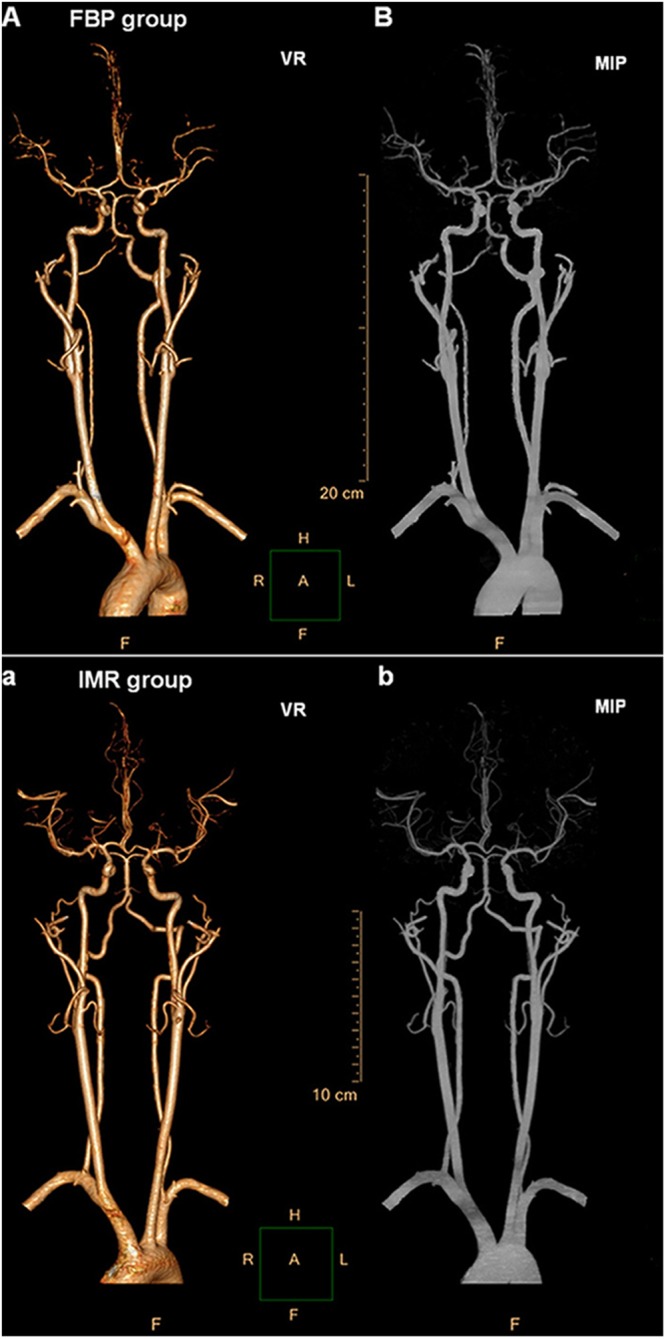
Measurements of the subjective image quality in the head and neck CTA of two 12-year-old boys reconstructed with the FBP algorithm and the IMR algorithm. (**A**,**B**) show the VR and MIP images of the FBP group, while a and b show the VR and MIP images of the IMR group. The subjective image qualities of both groups were rated as excellent (p < 0.01). The IMR group obtained a better image quality than that of group A (p < 0.01). Abbreviations: VR, volume rendering; MIP, maximum intensity projection.

The subjective image quality of group B, which was rated by two radiologists, was excellent (*κ* = 0.852, *p* < 0.01) (Figs 2 and 3). Both groups exhibited adequate image quality for diagnostic performance. Group B obtained a better imaging quality score (4.5 ± 0.6) than that of group A (3.9 ± 0.7) (Z = 3.517, *p* < 0.01) (Fig. 1).

## Discussion

According to the recommendations of the International Commission on Radiological Protection (ICRP)^22^, the protection of radio-sensitive organs, such as the eye lenses and thyroid gland, in head and neck CTA scanning is given top priority. In particular, it is meaningful to reduce the radiation dose in head and neck CTA for pediatric patients^4,5^.

Our current results demonstrated that CT imaging with the IMR algorithm in head and neck CTA enabled a significant reduction in patient radiation dose. Using IMR combined with a lower tube voltage (80 kV) and less iodinated CM (30 ml), the mean ED and iodine load of group B were reduced by 69.8% and 40.0%, respectively, compared to that of group A, which used the traditional FBP method combined with the normal tube voltage (100 kV) and iodinated CM (50 ml). In addition, the administered CM received by group B was reduced by 40% compared to that of group A. In theory, such dose reductions and lower CM intake might decrease the potential risks of ionizing radiation exposure, including radiation-induced malignancy^3^.

In addition to the dose reductions and lower CM intake, the IMR algorithm also guarantees a better image quality than that of the traditional FBP method. For the objective image quality, our results demonstrated that the IMR group exhibited an increased CT value, better SNR/CNR, and less image noise than that of the FBP group. For the subjective image quality, the IMR group obtained better imaging quality scores than those of the FBP group. It is known that reduced image noise aids in improving lesion detection and characterization, and this is especially beneficial for children, allowing for the detection of small lesions and improving lesion delineation.

Low tube voltage CT scan is proven to be a promising strategy to reduce the radiation dose and iodine load because of the greater photoelectric effect and reduced Compton scattering^23–25^. Despite the lower CM dose used in our current study, IMR, combined with the lower tube voltage, also increased the CT value compared to that of the FBP group. These results are consistent with previous studies of low tube voltage, which show increased CT values in the vessels^26,27^. Moreover, the excessive volume of the CM is closely related to CM-induced nephropathy and a higher extravasation rate^24,28,29^. Therefore, the strategy of a lower tube voltage, combined with reduced CM intake, is beneficial to children with renal disease or renal dysplasia and may help to alleviate safety worries for children.

However, a low tube voltage scan usually increases the image noise and, therefore, degrades the image quality due to the X-ray low-dose efficiency^30^. As a frequently used method, FBP reconstructs images from multi-angle images but limits further reducing the radiation dose and shows a deficiency in the image quality^14^. ASiR uses statistical models to reduce the radiation dose by 32–65%^15,31^, and MBIR uses a geometrical model to reduce image noise and improve spatial resolution^31^. However, it should be noted that the ASiR algorithm induces artifacts in the organization conjunction^18^ and the MBIR algorithm results in a slightly blurring of the imaging texture and induces a seriously delay in the reconstruction process^19^. The results of our current study demonstrated that the IMR technique in head and neck CTA could bring a significant reduction in the radiation dose, with an excellent image quality and good vascular delineation. For children, this method might provide an excellent solution for small vessels with minute lesions.

There are several limitations to our current study. First, despite the fact that we made every effort to enroll pediatric patients, the number of patients included in the current study was limited. Second, the CM intake in our current study was not the lowest, and the method how to determine the minimum CM intake must be further studied. Third, it is known that the low tube voltage tends to decrease the image quality for overweight or obese patients. However, our current study only included children with a normal or lower BMI. In addition, our study ignored the existing differences in the age of the enrolled patients, which could also introduce bias. Further studies with larger sample sizes and sub-group analyses, with different age and body size, are required to validate our strategy.

The current study was the first to evaluate the performance of IMR combined with a low tube voltage and a low iodine load in head and neck CTA for children. Our results demonstrated that the use of IMR enabled a reduced dose, with improved image quality. These results are in accordance with previous studies reported by Zhang *et al*.^21^ and Faggioni *et al*.^25^, and is of vital importance for clinical application in low dose head and neck CTA of pediatric patients. Further studies are required to verify how IMR affects the image quality in CT scanning in other parts of the body and whether further radiation dose reductions can be achieved under the guarantee of image quality and diagnostic capability.

## Methods

This study was approved by the local ethical committee of the Second Affiliated Hospital of Soochow University. The study protocols were performed in accordance with the approved guidelines and regulations. A total of 91 consecutive pediatric patients were enrolled in our current study from January 2016 to January 2017, and 83 participants (42 males/41 females, age range from 10 to 16 years old) met our inclusion criteria and received a head and neck CTA scan. Informed consent documents were obtained from all the enrolled participants and their legal representatives.

### General Information

For the inclusion criteria, children who suffered from suspected cerebral ischemic diseases were eligible for our study, which included headache, dizziness, nausea, vomiting, numbness, weakness or obstacles, walking instability, and speech problems. The exclusion criteria were as follows: (1) patients with severe heart, liver or renal insufficiency; (2) allergy to iodine CM; (3) prior surgery with intracranial implants, aneurysm clipping or embolization; and (4) a BMI ≥ 25 kg/m^2^.

### Imaging acquisitions

All the examinations were performed with a 256-slice CT (Brilliance iCT, Philips Healthcare, Cleveland, OH, USA). The CT scanning parameters were as follows: helical mode with 0.9 mm continued section thickness; layer spacing 0.45 mm; tube current 150 mAs; detector width 128 × 0.625 mm; pitch 0.992; rotation time 0.5 s; FOV: 250 × 250 mm; and matrix size 512 × 512.

All the patients were randomized into two groups. There were 42 patients in group A and 41 patients in group B. Group A used FBP image reconstruction combined with a 100 kV tube voltage and 50 ml iodinated CM (iopromide, 300 mg/ml; Ultravist, Bayer Schering Pharma, Berlin, Germany). Group B used IMR image reconstruction combined with an 80 kV tube voltage and 30 ml iopromide. Each group was injected with an injection rate of 5 ml/s and was followed by a 30 ml saline flush. The scanning scope arose from the arch of the aorta to the calvarium. After scanning, all the head and neck CTA original images are transferred to the workstation (Extend Brilliance Workspace V6.0.1, Philips Healthcare). The volume rendering (VR), maximum intensity projection (MIP) and curved planar reconstruction (CPR) images were obtained and evaluated by the vascular analysis software.

### Image reconstruction

All raw data were reconstructed using identical parameters of 0.9 mm thickness at 0.45 mm increment. The raw data from group A were reconstructed with an FBP algorithm, and the raw data from group B were reconstructed with IMR, level 1.

### Radiation Dose

The CTDIvol (mGy) and DLP (mGy·cm) were automatically generated by the system. The ED (mSv) was calculated as ED = DLP × k. k is the conversion factor. In the head and neck CTA scanning, the value of k was set at 0.0042 mSv/(mGy·cm)^15,32^. I (mg) = CM concentration (iodine content mg/ml) × CM dose (ml). The IDR was calculated as the CM concentration multiplied by the injection rate.

### Image quality evaluation

#### Objective evaluation

Following the random and double-blind method, two senior radiologists (visiting staff) reviewed and evaluated the images (including the objective and subject evaluation) of all the patients independently. Three circular ROIs were selected bilaterally for each patient, which were the origin of the CCA (7 mm^2^), the origin of ICA (7 mm^2^) and the M1 segment of the MCA (3 mm^2^) (Fig. 2). Then, we (1) measured the enhanced CT value (CTv) of the ROIs, avoiding touching the vascular wall and calcified plaque. The image noise was defined as the standard deviation value (SDv) of the ROIs. (2) We measured the mean CT values (CTm) of the bilateral paravertebral muscles or the temporal muscle of the abovementioned ROI levels. (3) The SNR and CNR were calculated by the following formulas: SNR = CTv/SDv and CNR = (CTv − CTm)/SDv.

#### Subjective evaluation

The subjective evaluation of the image quality (vascular delineation of arterial vessels, visibility of small arterial detail and image artifacts), lesion detection, and normal structure visualization followed the double-blind method and was performed using the 5-score method. The details of the 5-score method were as follows: 5-excellent image quality and contrast ratio, good vascular delineation, no artifacts, easy to diagnose; 4-good image quality and contrast ratio, normal vascular delineation, with a few artifacts, but adequate to diagnose; 3-satisfactory image quality and contrast ratio, some artifacts, the artery was not clearly displayed but was sufficient for the diagnosis; 2-weak image quality and contrast ratio, obvious artifact, the artery was not clearly displayed and was not sufficient for the diagnosis; 1-poor image quality and contrast ratio, severe artifacts, very hard to distinguish the small artery and could hardly make a diagnosis. More than 3 scores of the image quality were considered for the clinical diagnosis^30^. An example picture of the 5-score method used to evaluate subjective image quality is provided in the supplemental material (Supplemental Figure).

#### Statistical analyses

The demographics, clinical variables, scanning length and the hyoid level maximum diameter were assessed using Statistical Product and Service Solutions (SPSS, version 20.0; IBM, Armonk, New York, USA). All the data were expressed as the mean ± SD. A two independent sample t-test was used to compare the two groups for the CT value, CTDIvol, ED, signal noise, SNR and CNR. For the imaging quality scores, the Mann–Whitney U test was used for the analysis between the two groups. For the assessment of the subjective image quality, the inter-reader agreement was calculated using Cohen’s kappa statistic. Kappa values less than 0.20 were interpreted as poor agreement; 0.21–0.40, as fair; 0.41–0.60, as moderate; 0.61–0.80, as good; and 0.81–1.00, as very good agreement^18^. *p* values less than 0.05 were considered statistically significant.

#### Data Availability

The datasets generated during and/or analyzed during the current study are available from the corresponding author upon reasonable request.

## Electronic supplementary material

Supplemental figure
