# Supplementary material for: Impact of iterative model reconstruction combined with dose reduction on the image quality of head and neck CTA in children
Source: Sci Rep. 2018 Aug 22;8:12613. doi: 10.1038/s41598-018-30300-4 (PMC6105670; doi:10.1038/s41598-018-30300-4)
Supplement: Supplementary file 1 — Supplemental figure [file 41598_2018_30300_MOESM1_ESM.pdf]

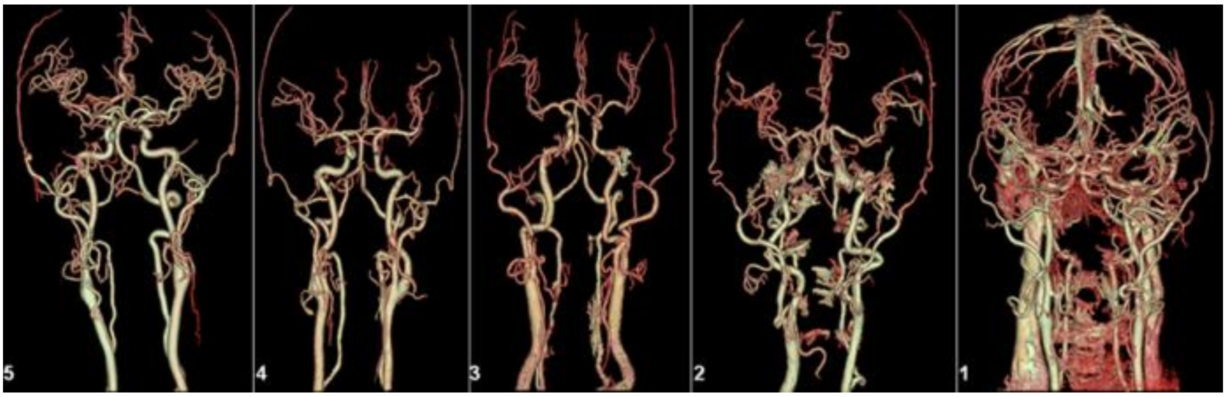

Supplemental figure. 5-score method used to evaluate subjective image quality

Impact of iterative model reconstruction combined with dose reduction on the image quality of head and neck CTA in children

Bochao Cheng, MD, PhD[1,2], Haoyang Xing, PhD[2,3], Du Lei, PhD[4], Yingkun Guo, MD[1,5], Gang Ning, MD[1], Qiyong Gong , MD, PhD[2] and Wu Cai, MD[6*]

Author Affiliations:

1.Department of Radiology, West China Second University Hospital, Sichuan University, Chengdu, China,

2.Huaxi MR Research Center, Department of Radiology, West China Hospital of Sichuan University, Chengdu, China,

3.College of Physical Science and Technology, Sichuan University, Chengdu, China.

4.Department of Psychosis Studies, Institute of Psychiatry, Psychology&Neuroscience, King's College London, London, UK.,

5.Key Laboratory of Birth Defects and Related Diseases of Women and Children (Sichuan University), Ministry of Education, Chengdu, China,

6.Department of Radiology, Second Affiliated Hospital, Soochow University, Suzhou, China,

[*]**Correspondence Authors:**

Wu Cai, Professor

Tel/Fax: +86 (0)512-67783438.   E-mail: bfviphx@163.com

Department of Radiology, Second Affiliated Hospital, Soochow University, #1055 Sanxiang Road, Suzhou, 215004, China
